# Lesions of the nucleus accumbens core modulate development of matching behavior

**DOI:** 10.1186/1471-2202-15-55

**Published:** 2014-04-30

**Authors:** Nobuyuki Kai, Yuji Tsutsui, Kazuto Kobayashi

**Affiliations:** 1 Department of Molecular Genetics, Institute of Biomedical Sciences, Fukushima Medical University School of Medicine, Fukushima 960-1295, Japan; 2 Faculty of Symbiotic Systems Science, Fukushima University, Fukushima 960-1296, Japan; 3 Present address; Department of Histology & Neurobiology, Dokkyo Medical University School of Medicine, Tochigi 321-0293, Japan

**Keywords:** Matching behavior, Nucleus accumbens core, Excitotoxic lesion

## Abstract

**Background:**

The development of choice is a crucial determinant in the performance of appetitive responses. Given two options with different reinforcement rates, animals match their relative rate of responding to the relative rates of reinforcement (i.e., matching behavior). A previous study has shown that the nucleus accumbens core (AcbC) is involved in the performance of matching behavior in trained animals. However, the role of the AcbC in the acquisition of matching behavior has not been addressed.

**Results:**

We conducted a series of experimental sessions to examine the role of the AcbC on the development of matching behavior. Instrumental responding was measured in rats with excitotoxic lesions of the AcbC. Rats were given two options that differed in the relative rate of reinforcement under concurrent variable-interval schedules. The locations of the more frequently reinforced option and the alternative option were randomly switched between sessions. Lesions of the AcbC accelerated the development of matching behavior compared to the sham-operated group. The AcbC-lesioned rats exhibited closer conformity to the matching law than shams when the options were in the same positions as in the previous session (the *same* condition), but not when the option locations had been switched (the *different* condition). The AcbC rats showed smaller probabilities of switching behavior between alternatives than shams. Post-reinforcement pausing was not affected by the AcbC lesion. Neither numbers of rewards obtained nor number of lever presses were different between the AcbC-lesioned rats and shams over session blocks.

**Conclusions:**

Our results suggest that the AcbC plays a regulatory role in the development of matching behavior through switching probabilities rather than perception of reward magnitude. The differential effect of AcbC lesions on the matching behavior between the *same* and *different* conditions suggests influence of the spontaneous recovery, that is, reversion to a previously reinforced choice at the beginning of the next session, on the development of matching behavior in the AcbC-lesioned rats.

## Background

In behavioral sciences, many studies have been conducted to investigate how animals select actions between multiple options with uncertain outcomes [1-3]. In a situation where two or more response options (e.g. levers) provide different rates of reinforcement, an animal’s relative response rates on the different options generally conform closely to the relative rates of reinforcement delivered by the options [4-6]. This assignment of response rate is known as matching behavior. Trained animals, with considerable previous experience in the different reinforcement rates of the options, are quick to adapt to unpredictable changes in relative reinforcement rates [7-17]. However, few attempts have been made to explore the patterns of acquisition of matching behavior in experimentally naïve animals [18,19].

The nucleus accumbens core (AcbC) is known to participate in behavioral tasks requiring an assessment of possible actions that differ in outcome value, such as risk-based decision-making [20,21], temporal discounting [22-24], and effort-based decision-making [25-28]. In addition, Cardinal and Cheung [29] reported that well-trained rats with excitotoxic lesions of the AcbC exhibited better matching behavior, potentially as a result of increased sensitivity to reinforcer magnitude. However, it is unknown how the AcbC is involved in the development of matching behavior.

In the present study, we examined the influence of bilateral excitotoxic AcbC lesions on the development of matching behavior. Lesioned rats were exposed to daily sessions with concurrent variable-interval (VI) schedules on two levers. During the VI schedule for a given lever, a reinforcer (food pellet) was delivered if the lever was depressed after a specific time interval following the previous reinforcer. The time interval varied randomly around an average for each lever. The allocation of the two VI schedules to the levers was randomly alternated between sessions. The deviation from matching [30] was measured for each daily session as an index of the development of matching behavior. These data were averaged for each two consecutive sessions (to give two-session blocks) before analysis.

## Results

### Histological assessment of AcbC lesions

**Figure 1:**
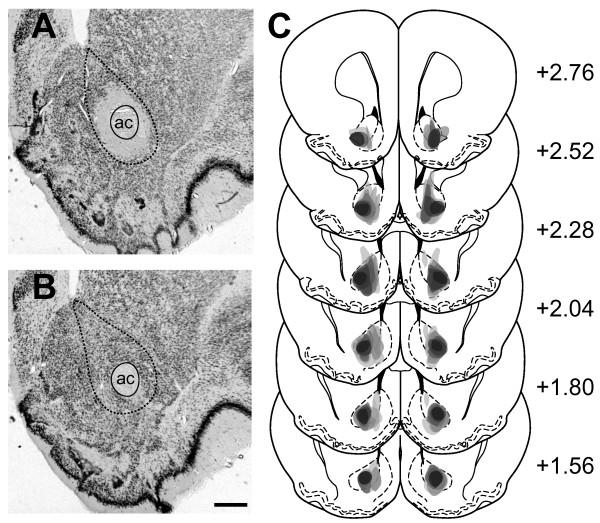
**Lesions of the AcbC. (A and B)** Photographs of coronal sections through the nucleus accumbens immunostained for NeuN from an AcbC-lesioned **(A)**, and a sham-operated **(B)** rat. The black dotted lines delineate the AcbC. The solid lines outline the boundary of the anterior commissure to facilitate its distinction from the area of lesion. Scale bar in B applies to all photographs and represents 0.5 mm. Abbreviation: ac, anterior commissure. **(C)** Schematic diagram of a series of coronal sections of the rat brain illustrating the extent of bilateral AcbC lesions (darkness represents coincidence of lesions from different animals). The numbers refer to millimeters anterior to bregma according to the standard atlas [39].

Verification of the lesion was made on brain sections that were immunostained for the neuronal nuclear protein NeuN. Ibotenic acid injection to the AcbC induced a marked neuronal loss around the injection site, observable as an unstained region (Figure 1A). The sham-operated rats showed no signs of cell loss (Figure 1B). The lesioned area extended anteroposteriorly from approximately 2.7 mm to 1.5 mm anterior to bregma, and did not extend ventrally or caudally into the olfactory tubercle or ventral pallidum (Figure 1C).

### AcbC-lesioned rats show altered matching behavior

**Figure 2:**
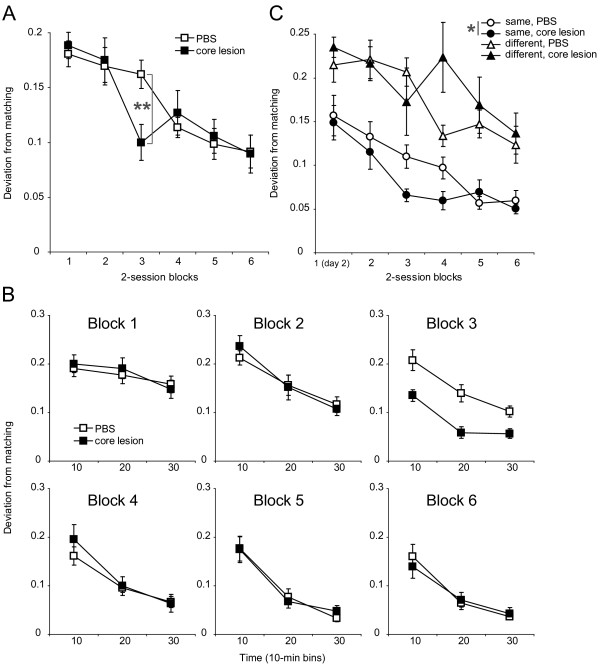
**Effect of the AcbC lesion on development of matching behavior. (A)** Data are expressed as mean (±SEM) of deviation from matching in the session block. Asterisks indicate a significant simple main effect of group (p < 0.01). **(B)** Each panel shows data from a particular session block (indicated at the top left of each panel). Data are expressed as mean (±SEM) of deviation from matching in the 10-min bins. The simple main effect of time (*p* < 0.001) is as follows: block 2, *F*(2, 44) = 42.469, ϵ = 1.0; block 3, *F*(1.7, 37.8) = 31.003, ϵ = 0.858; block 4, *F*(1.3, 29.4) = 34.219, ϵ = 0.667; block 5, *F*(1.4, 31.8) = 46.863, ϵ = 0.724; and block 6, *F*(1.5, 33.0) = 51.772, ϵ = 0.748. **(C)** Effect of the AcbC lesion on development of matching behavior across session blocks under the *same* and the *different* conditions. Data are expressed as mean (±SEM) of deviation from matching in each of the session blocks. *p < 0.05.

Deviation from matching behavior [30] was used as the performance index for the behavior. There was a clear decrease in the deviation across session blocks in both groups (Figure 2A). A three-way mixed analysis of variance (ANOVA, group × block × time) indicated a significant main effect of block [*F*(5,105) = 15.159, ϵ = 1.0, *p* < 0.001], with a significant block × group interaction [*F*(5,105) = 2.339, ϵ = 1.0, *p* = 0.047]. Post-hoc analyses of the group effect over session blocks revealed a significant simple main effect in the third session block [*F*(1,21) = 9.172, *p* = 0.006], in which the AcbC-lesioned rats exhibited closer conformity to the matching law than the sham-operated rats. Further, simple effects analyses revealed a significant effect of block in both the AcbC-lesioned [*F*(5,50) = 7.496, ϵ = 1.0, *p* < 0.001] and the sham-operated [*F*(5,55) = 10.446, ϵ = 1.0, *p* < 0.001] groups, confirming the development of matching behavior across session blocks in both groups.

We analyzed whether matching behavior would be improved within a session. The improvement of matching behavior across 10-min bins in the first session block was less evident than that on the second session block (Figure 2B). The overall ANOVA showed a significant main effect of time [*F*(1.8, 38.2) = 176.695, ϵ = 0.911, *p* < 0.001] with a significant time × block interaction [*F*(7.0, 147.9) = 3.66, ϵ = 0.704, *p* < 0.001], reflecting the differential improvement across session blocks. There were no significant interactions between time and any terms involving group in the same ANOVA (all *F* < 1). Post-hoc analyses conducted over each session block revealed that a simple main effect of time was significant in the first session block [*F*(1.8, 40.1) = 3.845, ϵ = 0.911, *p* = 0.033], as well as in the second session block onwards (*p* < 0.001; see legend of Figure 2B for each *F* value). These results indicate that matching behavior improves across 10-min bins over all session blocks in both groups.

The closer conformity to the matching law in the AcbC-lesioned rats of the third session block suggests that the AcbC may inhibit the improvement of matching behavior. In this study, the locations of the more frequently reinforced (rich) lever and the alternative (lean) lever were randomly alternated between sessions. The improvement of matching behavior is known to be delayed when the locations of the rich and lean levers are exchanged [14-16]. To test whether the AcbC lesions affected the responses to the change in lever location, the data were categorized into two conditions: one in which the locations of the rich and lean levers were the same as those in the preceding session (the *same* condition), and one in which the locations of the two levers were switched (the *different* condition). In the *same* condition, both the sham- and the AcbC-lesioned rats developed matching behavior as they experienced more sessions, and the AcbC-lesioned rats showed closer conformity to the matching law than the sham-operated rats (Figure 2C). A factorial 2 × 2 × 6 ANOVA with condition (*same*, *different*), group (AcbC lesion, sham) and session block (first to sixth, the very first session was excluded from the analysis) revealed a significant condition × group interaction [*F*(1,229) = 5.106, *p* = 0.025], while there was no interaction involving the block variable. Further analyses revealed that a simple main effect of group was significant under the *same* condition [*F*(1,135) = 6.054, *p* = 0.015], but not under the *different* condition [*F*(1,114) = 2.02, *p* = 0.158]. These results showed that the AcbC-lesioned rats exhibited closer conformity to the matching law than the sham-operated rats under the *same* condition but not under the *different* condition. Additionally, the statistical analysis revealed a simple main effect of condition in the AcbC-lesioned animals [*F*(1,109) = 75.385, p < 0.001], as well as sham-operated controls [*F*(1,120) = 56.605, p < 0.001]. This suggests that matching was better within either group in the *same* condition than in the *different* condition.

### AcbC-lesioned rats show altered switching behavior

**Figure 3:**
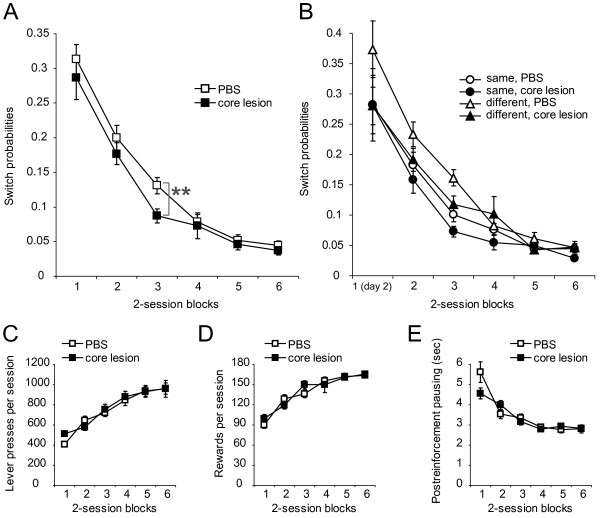
**Effect of the AcbC lesion on lever-press switching, performance, and pausing. (A)** Mean (±SEM) switching probabilities of lever presses between the AcbC-lesioned rats and shams over session blocks. Asterisks indicate a significant simple main effect of group (p < 0.01). **(B)** Comparison of switching between the *same* and *different* conditions. **(C)** Mean (±SEM) number of lever presses performed by the AcbC-lesioned and the shams over session blocks. **(D)** Number of pellets earned by each group in each session. **(E)** Post-reinforcement pausing between the AcbC group and shams. Data are expressed as mean (±SEM) pausing time in each session block.

To gain further insight into the underlying reason for improved matching behavior in the AcbC-lesioned rats, we analyzed switching probabilities. Subjects can switch their responses from one option to another under concurrent schedules, and probabilities of switching are known to be reduced by AcbC lesion [29]. We compared switching probabilities between the AcbC-lesioned group and the sham-operated group across sessions. AcbC-lesioned rats exhibited a smaller probability of switching than shams on session block 3 (Figure 3A). A three-way mixed ANOVA (group × block × time) revealed a significant effect of block [*F*(2.7,55.7) = 116.1, ϵ = 0.451, *p* < 0.001] and a significant group × block × time interaction [*F*(4.5,94.6) = 2.837, *p* = 0.024], while a main effect of group was not reliable [*F*(1,21) = 1.993, p = 0.173]. Further analyses confirmed the inspection of Figure 3A through a significant simple main effect of group [*F*(1,21) = 8.283, *p* = 0.009] in session block 3. From these results, we conclude that switching behavior is impaired by AcbC lesion in the third session block. We also carried out a three-way factorial ANOVA using factors of condition, separating the two lever locations by means of whether they were the same as or different to those in the preceding session, of block, and of group. The analysis revealed a significant main effect of group [*F*(1,229) = 9.202, *p* = 0.003], of block [*F*(5,229) = 100.762, *p* < 0.001], and of condition [*F*(1,229) = 16.163, *p* < 0.001]. No interactions including any variables were significant in the analysis. Thus the statistical analysis provided no evidence for a lesion effect on the switching behavior between the *same* and the *different* condition (Figure 3B).

### AcbC-lesioned rats do not show alterations in performance and pausing

We compared the number of responses, as well as rewards, between the AcbC- and sham-lesioned groups. Figure 3C shows averaged numbers of lever presses on both levers in each session block. The number increased as the experiment progressed in both groups. A three-way mixed ANOVA (group × session block × time) revealed a significant main effect of block [*F*(3.1, 64.2) = 73.779, ϵ = 0.611, *p* < 0.001]. There was no significant main effect or interaction involving the group variable. This result suggests that the AcbC lesions did not influence the overall performance of lever pressing. The number of rewards also increased in both groups across session blocks (Figure 3D). A three-way mixed ANOVA (group × session block × time) revealed a significant main effect of block [*F*(4.5, 94.4) = 56.613, ϵ = 0.899, *p* < 0.001]. There was no effect of group, and none of the interactions involving groups approached significance. This result indicates that the number of earned pellets was not affected by the AcbC lesions.

The improvement in conforming to the matching law in the AcbC-lesioned rats may suggest an exaggerated perception of reinforcer magnitude in these animals [29]. Post-reinforcement pausing (PRP) is defined as the time interval from the last delivery of the reinforcer to the next response emitted by a subject, and is jointly determined by past reinforcer magnitude and stimuli correlated with upcoming magnitude [31,32]. Dopaminergic innervation to the nucleus accumbens is known to be involved in the behavioral control of PRP [33,34]. To test whether the AcbC-lesioned rats display altered perception of the past and the upcoming reinforcer magnitude, we compared PRPs between the AcbC-lesioned rats and the sham-operated animals. PRPs declined across session blocks in both the AcbC-lesioned and the sham-operated animals (Figure 3E). An ANOVA using a between-subjects factor of group and a within-subject factor of session block showed a significant main effect of session block [*F*(1.9, 39.6) = 61.899, ϵ = 0.377, *p* < 0.001] with a significant group × session block interaction [*F*(1.9, 39.6) = 5.202, ϵ = 0.377, *p* = 0.011]. Simple effects analyses showed a significant effect of session block in both the AcbC-lesioned [*F*(2.7, 26.8) = 23.773, ϵ = 0.535, *p* < 0.001] and the sham-lesioned [*F*(1.5, 16.3) = 42.24, ϵ = 0.297, p < 0.001] rats. These results indicate that the AcbC-lesioned rats, as well as the sham-lesioned controls, can shorten PRPs by repetition of sessions. Post hoc comparisons of PRPs between the AcbC rats and shams failed to show any significant difference in all session blocks [in block 1 and 2, *F*(1,21) = 3.152 and 2.276, *p* = 0.09 and 0.146 respectively, *F* values were < 1 in block 3 and thereafter]. These results suggest that perceptions of the past and the upcoming reinforcer magnitude do not differ between the AcbC-lesioned rats and the sham-lesioned rats.

## Discussion

The present study demonstrates that the AcbC-lesioned rats develop matching behavior faster than the sham-operated rats, because AcbC-lesioned group in the third session block displayed closer conformity to the matching law than the sham group. More detailed analysis reveals that the AcbC rats exhibit the closer conformity to the matching law than shams when the location of the rich/lean levers remains the same between two successive sessions (the *same* condition), but not when the levers are switched between sessions (the *different* condition). As well as the deviation from matching behavior, switching probabilities are also contracted in the AcbC-lesioned rats. In contrast, the AcbC-lesioned rats do not show altered lever-press performance and post-reinforcement pausing (PRP). These results provide suggestions for possible roles of the AcbC on the development of matching behavior.

We found better matching in the AcbC-lesioned rats by means of manipulations of reinforcer frequencies. Cardinal and Cheung [29] report similar results through manipulations of reinforcer magnitudes. These studies demonstrate that the AcbC-lesioned rats are more sensitive than shams for comparative differences in the magnitude and frequency of reinforcers between alternatives. In the present study, however, the AcbC rats failed to show alteration of PRP. This finding suggests unaltered perception of the past and the upcoming reinforcer magnitude in these animals. In addition, the AcbC lesions influenced neither the number of lever presses nor the number of pellets earned. These results indicate that the perceptions of the absolute reinforcer magnitude are not different between the AcbC group and shams. Thus, we conclude that better matching of the AcbC-lesioned rats does not result from the exaggerated perception of reinforcer magnitude in these animals.

Both our study and a previous study [29] showed lower switching probabilities in AcbC-lesioned rats. These results raise a possibility that the reduced switching in the AcbC-lesioned rats leads their behavior choice to closer conformity to the matching law faster than shams. Reduction of switching rates generally represents an increase of the mean stay durations for levers. In the present study, however, the rich lever produced more frequent reinforcers than the lean lever. In this condition, we can speculate that the stay durations for the rich lever increased more than those for the lean lever in the AcbC-lesioned rats, resulting in the biased increment of stay durations for the rich lever in these rats. We suggest that this probably greater contrast of stay durations between levers in the AcbC-lesioned rats promotes faster development of matching behavior in AcbC-lesioned rats than shams in the present study.

This tendency of the AcbC-lesioned rats must be further strengthened in the *same* condition by an effect known as spontaneous recovery [14-16]. This means reversion to a previously reinforced choice proportion at the beginning of the session, for example, if the right lever is the rich lever in the current session, the rat will prefer pressing on the right lever at the start of the next session. Thus we speculate a reason for the better matching shown by AcbC-lesioned rats in the *same* condition, as both the lowered switching and the spontaneous recovery are additive for the development of matching behavior in this condition. Conversely, spontaneous recovery potentially interferes with the development of matching behavior in the *different* condition because the locations of scheduled levers (which lever is programmed to the VI 8 schedule) are exchanged between the current and the preceding session, and the percentage of responses at the beginning of the current session will be biased for the lever of the VI 72 schedule in this condition. Thus we speculate in the *different* condition that the spontaneous recovery cancels out the effect of reduced switching for faster development of matching behavior in the AcbC rats.

There are at least two possible mechanisms that may reduce the switching rate of the AcbC rats. First, the AcbC may be involved in the neural mechanism for the perception of changeover delay (COD), as previously suggested [29]. COD is a period of time following lever-press alternation during which reinforcement of responding on the lever cannot occur [4], and is set to be effective for 3 s in the present study. Switching rate in rats is known to be reduced when COD is increased [35], and the AcbC-lesioned rats are known to be sensitive for the delayed reinforcement [21-24,29]. Thus the potentially exaggerated perception of COD in the AcbC rats may reduce the changeover rate in these animals. Another possibility is impaired neural encoding for behavioral switching in the AcbC rats. Integrity of the nucleus accumbens is known to be necessary for both the acquisition and performance of cue-directed behavioral switching tasks [36,37]. The evidence for neural encoding for behavioral switching in the nucleus accumbens is provided by using neurophysiological recordings of well-trained rats during the cue-directed behavioral switching task [38], showing that nearly one third of the recorded neural responses were outcome-predictive with additional modulation by the presence or absence of a subsequent behavioral switching. From these reports, it is possible that some neurons in the AcbC encode switching information, and the loss of these neural responses might cause reduction of behavioral switching in the present study. The lower switching probability and better matching behavior in the AcbC-lesioned rats are due to both of the frequency (present study) and the magnitude (shown by [29]) in reinforcers. These observations suggest that the rate of behavioral switching is a mutual module of matching behavior to both of the frequency and the magnitude of reinforcers. Thus, the mechanism of AcbC-induced switching and its potential role on the development of choice should be further investigated.

## Conclusion

This study shows that AcbC lesions promote an improvement in conforming to the matching law and that the AcbC regulates the development of choice behavior. Switching behavior potentially mediates conformity to the matching law, and the reduced switching in the AcbC-lesioned rats suggests an effect of a primary change in switching behavior on the better matching of the AcbC-lesioned rats. Matching behavior of the AcbC-lesioned rats is also affected by the sequence of the left/right allocations of the more frequently reinforced lever in two successive session (the *same* or *different* condition) and is better than shams in the *same* condition but not in the *different* condition, suggesting influence of the spontaneous recovery on the development of matching behavior that is induced by the reduced switching in the AcbC-lesioned rats.

## Methods

### Subjects

Twenty-four male Long–Evans rats (Charles River Japan, Yokohama, Japan) weighing 450–500 g and aged 4 months at the beginning of the experiment were used as subjects. The rats were housed in groups of three in polycarbonate plastic cages located in a temperature- and humidity-controlled colony room under a 12-h light/dark cycle (lights on at 7:00 a.m.). During the experiment, rats were maintained on a restricted diet of 15 g lab chow per day. Water was available *ad libitum*, and food was given immediately after the daily experiment. All procedures were conducted in accordance with the guidelines established by the Animal Research Committee of Fukushima Medical University (Fukushima, Japan).

### Apparatus

Instrumental training and testing took place in four identical operant conditioning chambers (29.5 × 32.5 × 24.5 cm; Med Associates, East Fairfield, Georgia, VT) located in the colony room. The chamber was placed within sound- and light-attenuating housing equipped with a ventilation fan that also screened external noise. Inside the chamber, two (left and right) retractable levers (4.7 cm wide) were mounted on a panel 11 cm apart and 6 cm from the grid floor. A pellet dispenser (ENV-007CT, MED Associates) delivered individual 45 mg food pellets (Bio-Serve, Frenchtown, NJ) into a recessed magazine situated between the levers. The dispenser could be controlled manually by pressing a button on the outside of the cage. The chamber was equipped with a tone generator (ENV-230, MED Associates) and a speaker that was positioned 18 cm above the grid floor, on the wall opposite the levers. A white house light (2.5 W, 24 V) was also located on this wall. A charge coupled device camera was placed on the ceiling of the chambers to monitor the behavior of the animals. Control of the chambers and collection of the data were accomplished by MED-PC IV software (Med Associates) on a personal computer running the Windows XP operating system, which was connected to the operant chambers via a set of four interfaces.

### Initial training

Daily training sessions were conducted five days a week (Mon–Fri) between 8:00 a.m. and 2:00 p.m. At the beginning of each session, the house light was illuminated and the retractable levers protruded into the chamber. Under a continuous reinforcement schedule, in which each lever press was reinforced with the delivery of a food pellet, all rats learned to lever-press for food on both the left and the right lever. Each session ended after 30 min or 50 reinforcements, whichever occurred first. Pressing the levers resulted in the immediate delivery of a food pellet, during which a pure tone (10 KHz, 80 dB) sounded for 0.5 s. Some animals received a shaping procedure, in which approaching and touching the levers was reinforced with experimenter-driven delivery of a pellet. Successful lever training was defined as earning 50 food pellets within 5 min. All animals reached the criterion within eight sessions.

### Surgery

After initial training, all rats were randomly assigned to two groups that received bilateral injections into the AcbC of either 0.01 M phosphate-buffered saline (PBS; sham-operated group, *n* = 12) or ibotenic acid (Wako Pure Chemical Industries, Osaka, Japan; lesioned group, *n* = 12) dissolved in PBS (8 mg/ml). Injections were performed with the rats under sodium pentobarbital anesthesia (65 mg/kg i.p., Kyoritsu Seiyaku, Tokyo, Japan). The rats received injections of atropine sulfate (0.1 mg/kg i.p., Mitsubishi Tanabe Pharma, Osaka, Japan) and were placed on a stereotaxic apparatus (Narishige, Tokyo, Japan). The skull was exposed and two holes were drilled for the administration. The dura mater was broken with the tip of a hypodermic needle. We used borosilicate glass pipettes (outer diameter = 1.0 mm, Sutter Instrument Co, Novato, CA) pulled to tip diameters of 40 μm for injections into two sites per hemisphere. The coordinates were as follows [39]: (1) 2.2 mm anterior to bregma; ±1.6 mm lateral to the midline; and 6.5 mm below the brain surface; (2) 1.9 mm anterior to bregma; ±1.6 mm lateral to the midline; and 6.75 mm below the brain surface. Injection volume was 0.07 μl of ibotenic acid or PBS delivered to each injection site over 1 min with a micro-infusion pump connected to a 10 μl syringe (Hamilton, Reno, NV). The pipette was left in place for another 5 min to prevent spreading of the toxin along the capillary track. We resumed the food restriction 24 hours after the surgery. One rat that received ibotenic acid died following surgery, so 11 AcbC-lesioned rats and 12 sham-operated rats were used in the experiment.

### Testing

Sessions of the test phase began two weeks after the surgery and were conducted five days a week (Mon–Fri) between 8:00 a.m. and 2:00 p.m. We ran 12 daily sessions for 30 min under concurrent VI schedules on two levers. We set one VI schedule to deliver the reinforcer on an 8-s average interval and another on a 72-s average interval. The intervals for each schedule were derived from the formula developed by Fleshler & Hoffman [40]. For each animal, the allocation of the two VI schedules to each lever was randomly alternated with a probability of 0.5 after each session to discern the development of matching behavior from stimulus–response habit learning across sessions. There was no discriminative stimulus that signaled allocation of the VI schedules. At the beginning of each session, the house light was illuminated and the levers protruded into the chamber. The first response on each lever in each session was not always reinforced. As soon as an interval passed for either lever, pressing of that lever would deliver a reward. A changeover delay [4] was in place throughout sessions as a penalty for changing levers; a reward could not be obtained for 3 s after the first response on the new lever.

### Data analysis

All statistical analyses of the data collected were performed using the software Dr. SPSS II for Windows 11.0.1 J (IBM Japan, Tokyo, Japan) implemented on a personal computer running the Microsoft Windows XP operating system. We calculated absolute deviation from the matching law [4,5], as given by the following expression [30]:

$$\left|\frac{R_{1}}{R_{1}+R_{2}}-\frac{r_{1}}{r_{1}+r_{2}}\right|,$$

where *R*_*1*_ and *R*_*2*_ are responses to alternative levers, and *r*_*1*_ and *r*_*2*_ are obtained rewards on those alternatives. Therefore, if subjects were indifferent to both levers, the score of deviation would be 0.4 on average because an indifferent subject would allocate responses in a ratio of 0.5 and the reinforcement ratio would be approximately 8/(8 + 72) = 0.1. The deviation was calculated for 10-min bins in all sessions. Prior to statistical analysis, we took the mean for each two successive sessions (constituting a session block). Then, the data were analyzed by a 2 × 6 × 3 ANOVA with a non-repeated factor of group (AcbC lesion, sham lesion), and repeated measurement factors of session block (from the first to the sixth session block) and factors of time (three 10-min bins). For repeated measures analyses, Mauchly’s test of sphericity of the within-subject variables was applied and the Huynh-Feldt epsilon (ϵ) correction was used to evaluate *F* ratios for repeated measures involving more than one degree of freedom. Statistical significance was set at a probability level of *p* < 0.05 for all tests. Significant main effects or interaction terms were further investigated by post hoc comparisons using one-way ANOVA.

### Histology

After behavioral testing was complete, rats were anesthetized with a lethal dose of sodium pentobarbital (130 mg/kg) and perfused transcardially with PBS followed by 10% formalin neutral buffer solution (Wako). The brains were removed and postfixed in the same fixative for 24 h. Prior to sectioning, brains were transferred to 30% sucrose in 0.1 M phosphate buffer overnight at 4°C for cryoprotection. Coronal sections of 35 μm were cut on a freezing microtome (Leica Microsystems, Tokyo, Japan) and were processed for immunohistochemistry for the neuron-specific nuclear protein NeuN. Specifically, free-floating sections were incubated with 1% hydrogen peroxide in PBS for 30 min at room temperature (RT). After rinsing in PBS, sections were incubated with 5% normal swine serum (Vector Labs, Burlingame, CA) in PBS for 30 min at RT to block non-specific binding of primary antibody to the sections. They were incubated overnight at 4°C with a primary mouse anti-NeuN monoclonal antibody (Millipore, Billerica, MA, 1:400 dilution in PBS containing 0.3% Triton X-100). After rinsing, they were incubated for 2 h at RT with a secondary biotin-conjugated donkey anti-mouse antibody (Jackson Laboratory, Bar Harbor, MA, 1:1000) followed by another rinse. The bound antibodies were then visualized by an avidin-biotin-peroxidase complex system (Vectastain ABC Elite Kit, Vector) using 3,3-diaminobenzidine as the chromagen. All sections were mounted onto silane-coated glass slides and were coverslipped with Entellan New mounting medium (Merck, Damstadt, Germany).

## Abbreviations

AcbC: Nucleus accumbens core; ANOVA: Analysis of variance; ϵ: Huynh-Feldt epsilon; PBS: Phosphate-buffered saline; RT: Room temperature; SEM: Standard error of the mean; VI: Variable interval.

## Competing interests

The authors declare that they have no competing interests.

## Authors’ contributions

KN and KK conceived the study, designed the experiments, directed the project and drafted the manuscript. YT designed the operant response strategy and performed the behavioral analyses. All authors read and approved the final manuscript.
